# Clinical evaluation of atlas-based auto-segmentation for contouring pelvic CTVs in the treatment of anal cancer with FDG-PET-positive lymph node involvement

**DOI:** 10.3389/fonc.2025.1585338

**Published:** 2025-09-02

**Authors:** Max Bieder, Markus Böhm, Marciana-Nona Duma, Andrea Wittig

**Affiliations:** ^1^ Department of Radiotherapy and Radiation Oncology, Jena University Hospital, Friedrich-Schiller University Jena, Jena, Germany; ^2^ Institute for Medical Statistics, Computer Science and Data Science (IMSID), Jena University Hospital, Jena, Germany; ^3^ Department for Human Medicine, MSH Medical School Hamburg, Hamburg, Germany; ^4^ Department of Radiation Oncology, Helios Hospitals Schwerin, Schwerin, Germany; ^5^ Department of Radiotherapy and Radiation Oncology, University Hospital Würzburg, Würzburg, Germany

**Keywords:** atlas-based auto-segmentation, CTV auto-segmentation, anal cancer, PET/CT, lymph node metastases

## Abstract

**Introduction:**

Current evidence on atlas-based auto-segmentation (ABS) in radiotherapy primarily addresses organs at risk, whereas its application for clinical target volume (CTV) delineation remains insufficiently explored. Additionally, the optimal number of datasets required for ABS atlases is debated. This study investigates ABS performance for automated CTV (aCTV) segmentation in anal cancer patients with ^18^F-fluorodeoxyglucose positron emission tomography/computed tomography (^18^F-FDG PET-CT)-positive lymph node (LN) metastases, using varying atlas sizes.

**Methods:**

A retrospective analysis was conducted on 51 anal cancer patients who underwent ^18^F-FDG PET-CT-based treatment planning between 2009 and 2018. Patients with FDG-positive LN metastases were identified. Manual CTV (mCTV) delineation was performed in accordance with the UK National Guidance for IMRT in Anal Cancer. The resulting 51 mCTV datasets were integrated into a single ABS atlas, which was used to generate aCTVs for the 27 patients with FDG-positive LN metastases. For each of these 27 patients, five different atlas sizes (n = 10, 20, 30, 40, 50) were evaluated using a leave-one-out approach. Automated and manual CTVs were compared using the Dice Similarity Index (DSI), the percentage of FDG-positive LNs adequately covered, and volumes either erroneously included (mistakenly contoured volume, MCV) or omitted (not contoured volume, NCV) by the ABS process

**Results:**

Of the 51 patients, 27 (52.9%) had FDG-positive LN metastases. The mean DSI for atlas sizes of n = 10, 20, 30, 40, and 50 were 0.73, 0.78, 0.79, 0.79, and 0.80, respectively. A DSI ≥ 0.7 was achieved in 24 patients (88.9%) across all atlas sizes. The increase in DSI between n = 10 and n = 40 was statistically significant (Bonferroni-adjusted p < 0.05). Mean relative NCV and MCV ranged from 21.8–23.9% and 17.7–19.5% of the respective mCTV volume, with decreasing trends as atlas size increased. Segmentation inaccuracies predominantly occurred in the upper mesorectal and lower ischiorectal regions.

**Discussion:**

In conclusion, ABS facilitates the delineation of CTVs in anal cancer patients and improves contouring efficiency. However, manual correction by radiation oncologists remains necessary.

## 1 Introduction

Although anal cancer is relatively rare, its incidence has been increasing over the past two decades. The standard treatment involves combined chemoradiotherapy (1, 2). Accurate and efficient delineation of regions of interest (ROIs) is essential for radiotherapy planning, as precise calculation of the spatial dose distribution depends on objective and reproducible segmentation of both organs at risk (OARs) and tumor volumes, including the gross tumor volume (GTV) and the clinical target volume (CTV) (3). The CTV encompasses areas at risk of subclinical disease spread, such as lymphatic drainage regions, which are often not clearly visualized on planning computed tomography (CT) scans. Since prognosis in anal carcinoma is closely linked to lymph node (LN) metastases, accurate definition of the CTV and adequate dosimetric coverage of both macroscopic and microscopic disease are critical (4).

Manual delineation of ROIs is labor-intensive and time-consuming. Given the association between delays in radiotherapy and poorer clinical outcomes (5), there is a pressing need to streamline this process without compromising quality. This need is particularly acute in advanced techniques that require repeated planning, such as adaptive radiotherapy addressing anatomical changes throughout the treatment course.

Automated segmentation methods offer a promising alternative to manual contouring. A wide array of auto-segmentation techniques has been developed, aiming to enhance efficiency and reproducibility in the delineation of OARs. Early approaches involved basic image processing methods such as intensity thresholding, region growing, and heuristic edge detection (6). These were followed by region- and probability-based methods, and later by single-atlas and multi-atlas-based segmentation (ABS), the latter of which has become widely adopted in clinical practice (7).

More recently, deep learning (DL) models have been introduced and have demonstrated superior accuracy compared to ABS in ROI segmentation across various anatomical regions (8, 9). However, DL models require large, high-quality datasets (7), extended training times on high-performance graphics processing unit (GPU) clusters (10–12), and are susceptible to overfitting. Their complex, multilayered architectures complicate retraining, guideline updates, and reproducibility (6, 10). As a result, ABS remains the standard in many clinical workflows.

To date, most ABS studies have focused on the automated segmentation of OARs (13), with validation studies in anatomical sites such as the brain (14), head and neck (15), thorax (12, 16) and prostate (13), emphasizing segmentation accuracy and contour reproducibility. In contrast, automated segmentation of target volumes remains underexplored and more challenging, primarily due to tumor- or treatment-induced anatomical distortions. Automated tools often struggle to accurately segment the primary tumor region. However, the lymphatic drainage areas, particularly in cases without major anatomical alterations due to surgery or extensive lesions, are more consistently identifiable. In anal cancer, where such conditions are frequently met, ABS tools may be capable of reliably segmenting the pelvic lymphatic drainage pathways, and hence the CTV.

Nonetheless, auto-segmentation in the pelvic region presents specific challenges (17). The normal anatomy is highly variable, with organs such as the bladder, bowel, and genital structures exhibiting significant intra- and inter-patient variability in shape, filling status, and position (7, 18). Moreover, it remains uncertain whether atlas-based – therefore anatomically-based CTV segmentation adequately includes LNs that are involved but lack clear CT morphological criteria of malignancy. Therefore, Fluorodeoxyglucose positron emission tomography/computed tomography (^18^F-FDG PET-CT) is recommended during initial staging for the detection of LN metastases (19–21), offering high sensitivity (93%) and is considered a reference standard (22–25). The distribution of pathological and non-pathological LNs varies significantly between individuals, potentially leading to complex and unexpected anatomical patterns. Dapper et al. reported that approximately 20% of FDG-positive LNs in the inguinal, paraaortic, and common iliac regions were located outside of the areas covered by existing pelvic lymphatic delineation guidelines (26). This contributes to inter-observer variability and complicates consistent CTV definitions, particularly when evaluating automated segmentation performance.

To the best of the authors’ knowledge, only seven studies have addressed ABS-based CTV delineation in pelvic malignancies (17, 27–32). Of these, only one has performed a clinical validation of the ABS-generated CTV, and that study used an atlas derived from just four datasets (32).

The present study, therefore, has two main objectives: (a) to clinically validate an atlas-based CTV definition by comparing it to manually defined CTVs using established similarity metrics; and (b) to assess whether PET-positive LNs are adequately included within the atlas-based CTV contours, thereby evaluating its accuracy in encompassing metastatic involvement.

## 2 Materials and methods

Consecutive patients diagnosed with anal canal cancer or cancer of the anal verge between 2009 and 2018, who underwent PET-CT-based treatment planning, were retrospectively identified from the institutional database (N = 51). Patients with distant metastases (M1) were excluded. CT and PET-CT images were imported into the treatment planning system RayStation version 10B (RaySearch Laboratories, Stockholm, Sweden).

First, the CTV was manually delineated on the CT images using the Structure Definition module. A single observer performed all delineations based on anatomical landmarks in accordance with standard guidelines proposed by Muirhead et al. (33)^1^. Following these guidelines, the CTV included the following sub-volumes, each contoured as a separate ROI: external and internal iliac, obturator, presacral, inguinal, mesorectal, and ischiorectal regions. The ischiorectal space was delineated in all patients, and the obturator nodes were included within the internal iliac space.

Next, all sub-volume ROIs were combined into a single manually contoured CTV (mCTV) using the union function in the ROI algebra tool in RayStation. The resulting mCTV was then manually refined to correct for any anatomical irregularities.

Subsequently, PET-CT and planning-CT images were co-registered using RayStation’s Image Registration module for the identification of LN metastases in the 27 patients clinically staged as N1 or higher. PET images were rigidly aligned to the planning CT, and metabolic activity was assessed by overlaying PET signal on the CT anatomy using the Fusion view. LNs were classified as FDG-positive if they demonstrated increased FDG uptake and measured greater than 1.0 cm in size. These LNs were delineated in the Structure Definition module and included in the GTV as macroscopic disease. The delineated LNs collectively constituted the total LN volume ROI (Vt). The mCTV was subsequently expanded to include these FDG-positive LNs and surrounding areas to account for potential microscopic disease extension.

ABS was performed using RayStation’s dedicated ABS module. The atlas was constructed from planning CT datasets of multiple patients, each with a corresponding mCTV. To assess the effect of atlas size on segmentation accuracy, all 51 planning CT datasets with corresponding mCTVs were sequentially incorporated into the ABS atlas in a predefined order using the Structure Template Management tool: the first 27 patients with LN metastases were followed by the 24 patients without LN involvement. Auto-segmented CTVs (aCTVs) were generated using RayStation’s ABS algorithm, which applies rigid image registration (RIR) followed by deformable image registration (DIR) using the ANACONDA algorithm. This algorithm integrates both intensity-based and ROI-based information. Based on similarity metrics, the algorithm selected the most suitable atlas datasets (“fusion atlases”) and merged their segmentations into a consensus ROI. A leave-one-out approach was employed, excluding the target CT dataset from the atlas during each segmentation. Mesh-based structure adaptation (MBS) was enabled, and the number of fusion atlases was set to 15.

To investigate the influence of atlas size on segmentation performance, aCTVs were generated exclusively for the 27 patients with FDG-positive LN metastases using five different atlas sizes (n = 10, 20, 30, 40, and 50), where *n* refers to the number of CT datasets used during each segmentation. Because LN-positive patients were added first, the five atlas configurations included the following numbers of LN-positive patients: 10, 20, 27 (plus 3 LN-negative), 27 (plus 13 LN-negative), and 27 (plus 23 LN-negative), respectively.

**Figure 1:**
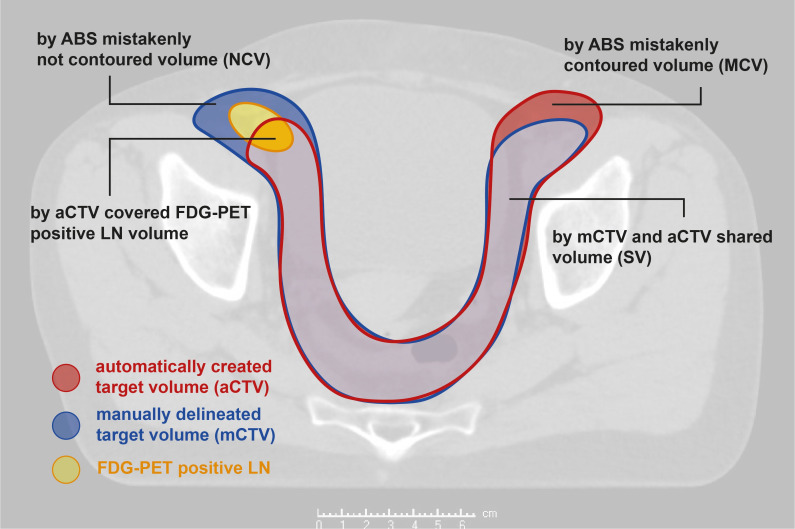
Considered volumes; aCTV, automatically generated clinical target volume; mCTV, manually delineated clinical target volume; LN, lymph node.

The resulting aCTVs were compared to the corresponding mCTVs using established similarity metrics (**Figure 1**). This analysis was performed specifically in the 27 patients with FDG-positive LN metastases. The shared volume (SV) between the mCTV and aCTV represented the correctly contoured region identified by the algorithm. Automatically segmented regions extending beyond the mCTV were categorized as mistakenly contoured volume (MCV), while areas of the mCTV not covered by the aCTV were defined as mistakenly not contoured volume (NCV). Both MCV and NCV values were calculated relative to the respective mCTV volumes, resulting in the relative mistakenly contoured volume (rMCV = MCV/mCTV) and the relative mistakenly not contoured volume (rNCV = NCV/mCTV).

The similarity of mCTVs and aCTVs was evaluated using the Dice similarity index (34):


$$DSI=\frac{2\times mCTV}{SV+\;aCTV}$$

SV – shared volume; mCTV – manually contoured target volume;

aCTV – automatically contoured target volume

A DSI of 1.00 corresponds to entire mutual covering of target volumes (value and position). Non-overlapping target volumes lead to a DSI of 0.00. In line with previous studies, a DSI of ≥0.70 was considered a threshold for clinical acceptable segmentation performance (13, 35). A LN ratio (Q_LN_) was defined as the amount of FDG-positive LN volume sufficiently covered by the aCTV [Q_LN_ = V_c_/V_t_ (V_c_ - *LN volume covered by aCTV; V_t_ – total LN volume)].* A Q_LN_ of 100.0% indicated a complete covering of PET-positive LN volumes while LNs were fully excluded from the aCTV if Q_LN_ was 0.0%.

For statistical analyses, SPSS^®^ Statistics Version 27 (IBM^®^, Armonk, NY, USA) was used. Differences between the five aCTVs regarding DSI, rMCV, rNCV and Q_LN_ were assessed using the Friedman Test. In cases of statistical significance (p-value <0.05), *post-hoc* analyses were performed using Dunn-Bonferroni tests to identify the specific values of n responsible for the observed differences. The Bonferroni correction was applied to adjust the alpha error rate, accounting for multiple comparisons following the Friedman test. The study was approved by the local ethics committee under Reg.-Nr. 2023-2909-Daten and consent was obtained from one patient for the publication of exemplary CT slices.

## 3 Results

51 consecutive patients were identified, who all underwent PET-CT based treatment planning. **Table 1** represents the patient characteristics.

**Table 1:** Patient characteristics.

	Characteristic	Count N	Percentage (%)
Gender	male	19	37.3
	female	32	62.7
Tumor localization	anal canal	28	54.9
	anal verge	19	37.3
	anal canal and anal verge	4	7.8
T-Stage	T1	10	19.6
	T2	19	37.3
	T3	15	29.4
	T4	6	11.8
	n.a.	1	2.0
N-Stage	N0	22	43.1
	N1	13	25.5
	N2	11	21.6
	N3	5	9.8
M-Stage	0	51	100.0
Grading	1	1	2.0
	2	35	68.6
	3	11	21.6
	4	1	2.0
	n.a.	3	5.9

**Figure 2:**
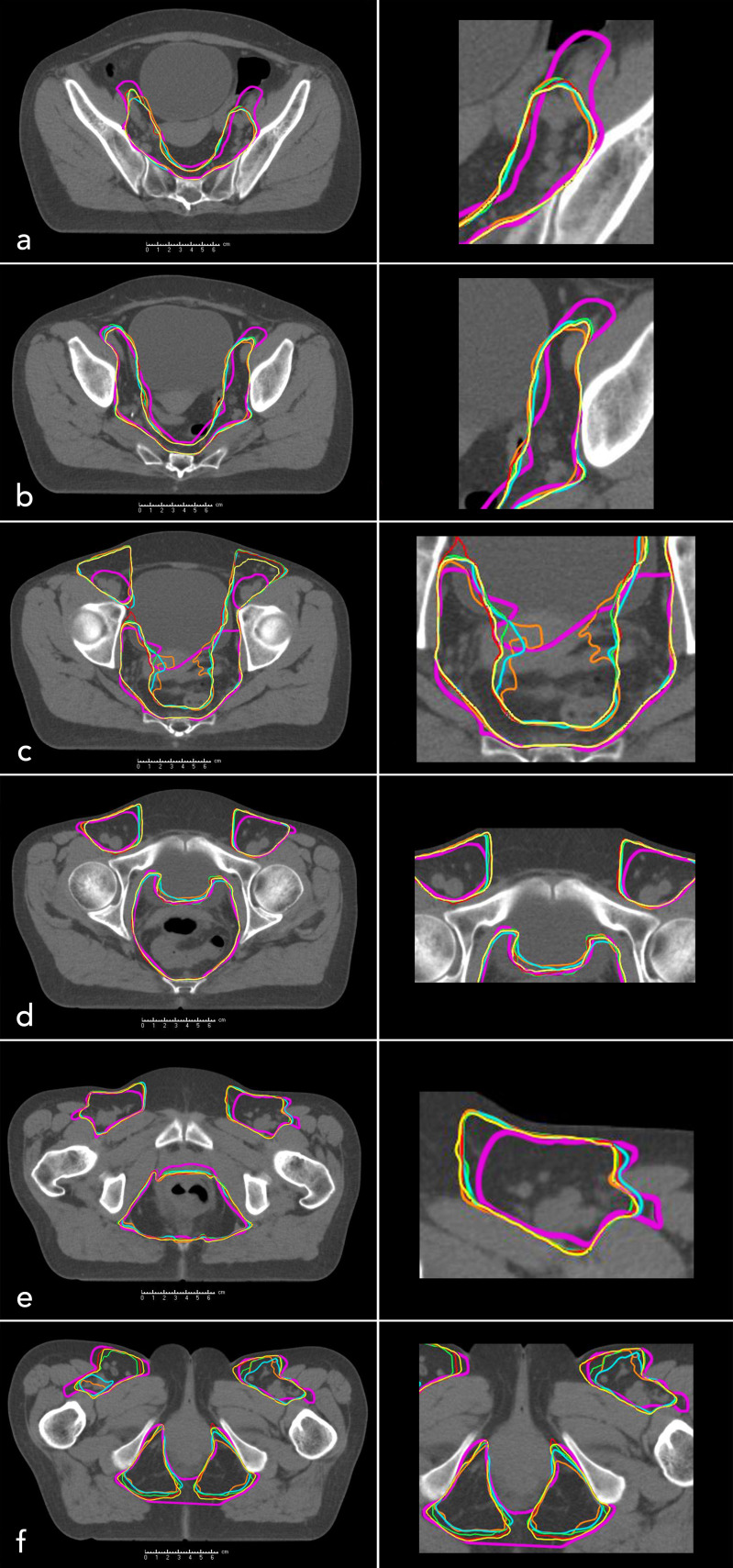
Manually contoured clinical target volume (mCTV, purple, patient 1) and automatically generated clinical target volumes for different atlas sizes: n = 10 (orange), n = 20 (blue), n = 30 (green), n = 40 (red), n = 50 (yellow). Axial CT slices of the pelvis are shown from cranial to caudal levels: **(a–f)**.

In the context of radiation planning, 29 patients were initially classified as FDG-positive (N-Stage ≥1). However, two of these patients showed doubtful FDG-positivity and were therefore reclassified as N-Stage 0 for this study. Consequently, 27 of 51 patients (52.9%) were considered FDG positive. Among these, a median number of two LNs showed an increased FDG-uptake. FDG-positive LNs were most frequently located in the inguinal region (77.8%, N=21), followed by the external and internal iliac (29.6%, N=8, respectively), the mesorectal (25.9%, N=7) and presacral space (18.5%, N=5). **Figure 2** provides a representative visualization of CTV contours across different atlas sizes, displayed on selected CT slices, highlighting the variations in segmentation accuracy as the atlas size increases.

**Figure 3:**
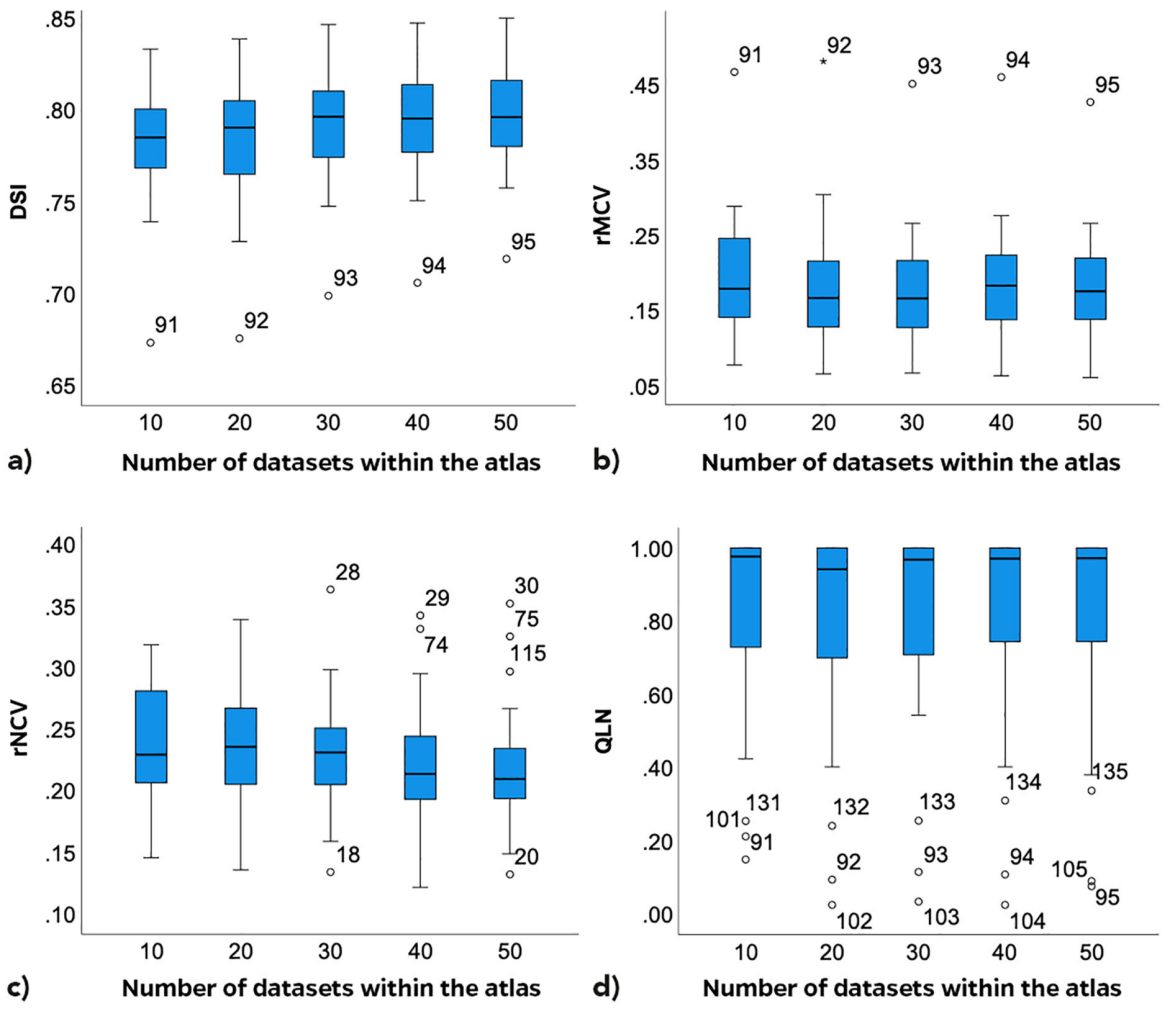
Boxplots depending on number n of datasets within the atlas; Dice similarity index – DSI **(a)**; related mistakenly contoured volume – rMCV **(b)**; related mistakenly not contoured volume – rNCV **(c)**; lymph node ratio – Q_LN_ **(d)**.

The DSI value of 0.18 for patient 2 with n=10 was identified as an outlier based on the deviation of the interquartile range. It was excluded from the analyses to prevent distortion of the results. The DSIs ranged from 0.67 (patient 19, n=10) to 0.85 (patient 16, n=50). The average DSIs for n=10, 20, 30, 40 and 50 amounted 0.78, 0.78, 0.79, 0.79 and 0.80, respectively. The median DSIs ranged from 0.79 (n=10) to 0.80 (n=30; 50) (**Table 2**). There was a statistically significant increase of the DSI between n=10 and n=40; n=50 (Friedman Test: p<0.05; *post-hoc*-test: Bonferroni adjusted p_1_ = 0.012; p_2_ = 0.006, respectively). **Figure 3A** depicts the boxplots for the different DSIs.

**Table 2:** Descriptive statistics of Dice similarity index (DSI).

DSI	Number n of datasets within the atlas template				
	10	20	30	40	50
Mean	0.73	0.78	0.79	0.79	0.80
95%-Confidence-Interval	[66.2-80.2]	[76.9-79.7]	[0.78-0.80]	[0.78-0.80]	[0.78-0.81]
Median	0.78	0.79	0.80	0.80	0.80
Standard deviation	0.18	0.04	0.03	0.03	0.03

The rMCVs varied from 6.1% (patient 6, n=50) to 48.1% (patient 19, n=20) (**Table 3**). The aCTV in patient 19 showed noticeable overextension beyond the intended anatomical regions in the upper mesorectal and lower ischiorectal spaces. The inguinal aCTV partially covered the psoas muscles laterally and dorsally. The boxplots of the five rMCVs (**Figure 3B**) depict a statistically significant decrease of the rMCV with increasing n (Friedman Test, p<0.01). Statistically significant differences between the rMCVs with n=10 to n=20; n=10 to 30; n=10 to 50 were identified (Friedman Test: p<0.05; *post-hoc*-test: Bonferroni adjusted p_1_<0.001; p_2_<0.001; p_3_ = 0.011; respectively). There was no statistically significant difference between n=10 and n=40.

**Table 3:** Descriptive statistics of relative mistakenly contoured volume (rMCV) and relative mistakenly not contoured volume (rNCV).

rMCV (%)	Number n of datasets within the atlas				
	10	20	30	40	50
Mean	19.5	18.3	17.7	18.2	18.3
95%-Confidence-Interval	[16.3-22.6]	[15.1-21.5]	[14.7-20.7]	[15.2-21.4]	[15.4-21.2]
Median	17.9	16.7	16.6	18.3	17.6
Standard deviation	7.8	8.1	7.5	7.8	7.3
rNCV (%)	Number n of datasets within the atlas				
	10	20	30	40	50
Mean	23.5	23.9	22.9	22.3	21.8
95%-Confidence-Interval	[21.8-25.2]	[22.0-25.9]	[21.0-24.7]	[20.3-24.3]	[19.9-23.8]
Median	22.9	23.6	23.1	21.3	20.9
Standard deviation	4.3	5.0	4.7	5.0	4.9

The rNCVs ranged from 12.1% (patient 4, n=40) to 36.3% (patient 6, n=30). The small rNCV in patient 4 was mainly due to a correctly contoured ventral inguinal aCTV. Sources of mistakes were the inguinal aCTV spaces in obese patients 6 and 15 (numbers 28–30 and 74–75 in **Figure 3**) as they were not sufficiently expanded ventrally to the recommended 5 mm from the skin surface (33). While the aCTV in the upper mesorectal and lower ischiorectal spaces was often overextended, this inconsistency also contributed to missed target volumes in adjacent or critical regions. The boxplots for the rNCV suggested a decreasing trend of rNCVs with increasing n (**Figure 3C**). A statistically significant decrease of the rNCV was found between n=10 to n=40; n=50 and from n=20 to n=40; n=50 (*post-hoc*-test: Bonferroni adjusted p<0.05, respectively). The median rMCVs were consistently smaller than the median rNCVs across all five atlas sizes (**Table 3**).

In 8 of the 27 patients with LN metastases (29.6%), the LN volume was completely covered by the aCTV, regardless of the template size. In two patients, an entire covering of the FDG-positive LN volume was achieved from n=30 (7.4%). The median Q_LN_ increased with the number of datasets after it passed a minimum at n=20 (**Figure 3D**). From n=20 to n=40, the Q_LN_ improved with statistical significance (*post-hoc*-test: Bonferroni adjusted p<0.05). Three patients (19, 21 and 27) were outliers with median Q_LN_ less than 40.0%. In Patient 19 (91-95, **Figure 3**), one presacral LN near the promontory and one mesorectal LN was not adequately covered because of an insufficient upper expansion of the mesorectal aCTV. In the CTs of patients 21 and 27 (101-105; 131-135, **Figure 3**), the aCTV was contoured correctly but was not expanded according to the individual requirements. In both patients, external iliac LN volumes were partially localized outside the recommended 7 mm medial to the external iliac vessels (33).

## 4 Discussion

While previous studies on ABS have predominantly addressed the delineation of OARs (13, 36–38), investigations focusing on the automated segmentation of entire CTVs remain limited. This study evaluated whether ABS can generate clinically acceptable CTVs for anal cancer, particularly in the context of PET-CT-identified LN metastases and explored the influence of atlas size on segmentation performance. Three main findings emerged: (1) the DSI significantly increased with growing atlas size, indicating improved segmentation accuracy; (2) median rMCVs were consistently smaller than rNCVs and decreased with larger atlas sizes; and (3) in the majority of cases, LN metastases were insufficiently covered by aCTVs, necessitating manual corrections.

Manual CTV delineation in radiotherapy planning is labor-intensive (39), and treatment delays are associated with an elevated risk of local recurrence (40). Moreover, manual segmentation introduces intra- and inter-observer variability (41, 42), which is exacerbated by the low soft tissue contrast and artifact susceptibility of planning CT scans, potentially affecting planning target volume coverage. These challenges may result in inadvertent irradiation of healthy tissue or incomplete tumor coverage (11, 41).

Automated segmentation methods offer potential solutions to these issues. ABS can expedite the workflow for radiation oncologists and has demonstrated time-saving advantages in delineating both OARs and CTVs (43). Additionally, consistent use of the same algorithm and atlas improves reproducibility and contouring consistency (44).

Several algorithmic approaches exist. Intensity-based methods classify voxels using criteria such as Hounsfield unit thresholds and are suited for regions with distinct contrast (11). Deformable shape models adjust predefined contours according to image data to generate ROIs (11). More recently, ABS and machine learning approaches—including both conventional and DL methods—have advanced significantly.

Comparative studies, such as one evaluating the DL-based software DLCExpert (Mirada Medical Ltd., Oxford, UK) versus ABS for OAR segmentation in head and neck, thoracic, and pelvic CTs, found DL to produce a greater proportion of clinically acceptable segmentations (8). Gibbons et al. reported that DL outperformed ABS in anatomically variable organs due to DL’s superior adaptability, achieved through large-scale datasets and millions of parameters (11, 12). However, DL requires extensive datasets and powerful computing infrastructure (e.g., GPU clusters) and is prone to overfitting when models become overly complex (10). Moreover, the opacity and limited reproducibility of deep neural networks hinder correction of systematic errors and adaptation to evolving guidelines. In contrast, ABS requires fewer datasets and can be run on standard workstations, rendering it a more cost effective, practical and widely adopted solution in current clinical settings (11).

The heterogeneous distribution of LN metastases in anal cancer complicates ABS-based CTV segmentation (26, 45, 46). Conventional ABS algorithms do not typically incorporate PET-CT data and are limited in adapting to highly variable anatomy (11). Nevertheless, ABS can encompass areas at high risk for LN involvement, and standardized atlases based on consensus guidelines may enhance segmentation accuracy. However, the degree to which current ABS algorithms adequately cover LN metastases remains uncertain.

In multi-atlas ABS, a library of segmented datasets serves as the reference. Image registration—typically based on mutual information—is used to map atlas ROIs to the target CT scan (11). Although segmentation accuracy generally improves with larger atlas sizes, computational costs also increase (37). Hence, identifying the optimal atlas size is essential. Current literature reports varying recommendations, depending on anatomical regions and ABS algorithms used (47). For example, Sjöberg et al. used 15 atlas datasets for pelvic LN segmentation and achieved median DSIs around 0.7 (48). Li et al. compared atlas sizes ranging from 20 to 120 for cervical cancer CTV and OAR delineation and found no significant differences between groups (36). Anders et al. stratified an atlas by patient sex and reported mean DSIs of up to 0.83 for automatically segmented substructures in anorectal cancer (35).

In our study, similarity metrics between aCTVs and mCTVs were in line with these findings. According to Anders et al. and Aoyama et al., a DSI > 0.7 reflects acceptable geometric similarity (13, 35). This threshold was met in 88.9% (24/27) of cases across all atlas sizes. Median DSIs ranged from 0.67 to 0.85. In a few instances (patients 2, 19, and 24), the threshold was not reached for certain atlas sizes.

Analysis of segmentation errors revealed consistent inaccuracies in the upper mesorectal and inguinal regions. Particularly in the mesorectum, anatomical variability and low tissue contrast complicate registration. In contrast, the internal iliac regions were segmented more accurately, likely due to their boundaries being defined by hyperdense pelvic bones, which favor automatic contouring.

Coverage of FDG-positive LN metastases by aCTVs was suboptimal, necessitating manual corrections. Manual CTV delineation was guided by the UK IMRT recommendations for anal cancer. Despite its accuracy in the study by Dapper et al., up to 20% of LNs remained uncovered in the common iliac, para-aortic, and inguinal regions (26). The absence of consistent recommendations—especially for inguinal coverage—likely contributes to these limitations in ABS.

Only 8 of 27 patients (29.6%) had complete LN coverage by aCTVs. In 2 additional cases (7.4%), increasing atlas size led to full LN inclusion. As noted in earlier studies (49, 50), the inguinal region, particularly its ventral boundary in obese patients, posed challenges due to ABS’s limited adaptability to patient-specific anatomy (11). Expanding the atlas with anatomical outliers may help address this issue. Insufficient cranial expansion of the CTV and LN locations outside the recommended 7 mm boundary of external iliac vessels also contributed to inadequate coverage, highlighting ABS’s current inability to incorporate PET-CT data. Despite guideline adherence, such discrepancies necessitate manual review and correction by radiation oncologists.

Our data indicate a progressive improvement in CTV quality with increasing atlas size. A median DSI of 0.80 was achieved with 30 datasets. Although no definitive optimal atlas size could be established, the consistent improvements up to 50 datasets suggest that larger atlases yield better results. This finding contrasts with Li et al. (36), who observed no significant improvement beyond 20 datasets in cervical cancer radiotherapy.

A limitation of this study is its focus on geometric metrics, which do not capture contour complexity (11). However, incorporating clinically relevant aspects, such as FDG-positive LN coverage, strengthens the analysis. Further research including dosimetric evaluations and OAR involvement is warranted. The small sample size limited the study to descriptive statistics.

The impact of atlas composition remains unclear. Although a leave-one-out approach was used for validation, the non-randomized sequential inclusion of datasets may introduce bias. In this study, LN-positive patients—representing more complex anatomies—were included first, leading to an uneven distribution of clinical phenotypes. Whether nodal status alone affects CTV variability or whether other factors, such as sex, pelvic morphology, or BMI, play greater roles remains to be determined. Future studies can benefit from subgroup-specific analyses to identify anatomical features that systematically affect segmentation accuracy. Nonetheless, segmentation quality improved even when LN-negative cases were added, supporting the robustness of larger atlases. Future studies should explore stratification strategies, such as phenotype balancing or subgroup-specific atlases.

Given that a commercial ABS platform was used, results may vary with different algorithms. However, algorithm-specific settings (e.g., number of fusion atlases) were optimized per manufacturer recommendations to ensure reproducibility and performance.

In conclusion, ABS can generate high-quality whole CTVs for anal cancer. In 88.9% of cases, acceptable DSI thresholds (≥0.7) were met across all atlas sizes, with performance improving alongside atlas expansion. However, persistent inaccuracies—particularly in anatomical outliers and LN coverage—necessitate manual adjustments. To improve robustness, ABS atlases should include anatomically diverse datasets reflecting different tumor stages and patient morphologies.

Compared to DL-based segmentation, ABS requires fewer datasets and allows more flexible adaptation to changing guidelines. Further studies are needed to determine the ideal atlas size, structure, and the potential benefits of incorporating anatomical outliers.

## Data Availability

The raw data supporting the conclusions of this article will be made available by the authors, without undue reservation.

## Footnotes

1 Note: An updated version of the national guidance for VMAT/IMRT in anal cancer was published by the Royal College of Radiologists in 2024. The present study was conducted in accordance with the 2016 guidance.
